# MicroRNA as a Potential Biomarker and Treatment Strategy for Ischemia-Reperfusion Injury

**DOI:** 10.1155/2021/9098145

**Published:** 2021-11-20

**Authors:** Mingming Cao, Wenjing Song, Runyu Liang, Lili Teng, Mei Zhang, Jiyao Zhang, Luwen Zhu

**Affiliations:** ^1^Heilongjiang University of Chinese Medicine, 24 Heping Road, Xiangfang District, Heilongjiang, Harbin 150040, China; ^2^Second Affiliated Hospital of Heilongjiang University of Chinese Medicine, 411 Guogeli Street, Nangang District, Heilongjiang, Harbin 150001, China

## Abstract

Ischemia-reperfusion (I/R) injury is a progressive injury that aggravates the pathological state when the organ tissue restores blood supply after a certain period of ischemia, including the myocardial, brain, liver, kidney, and intestinal. With growing evidence that microRNAs (miRNAs) play an important role as posttranscription gene silencing mediators in many I/R injury, in this review, we highlight the microRNAs that are related to I/R injury and their regulatory molecular pathways. In addition, we discussed the potential role of miRNA as a biomarker and its role as a target in I/R injury treatment. Developing miRNAs are not without its challenges, but prudent design combined with existing clinical treatments will result in more effective therapies for I/R injury. This review is aimed at providing new research results obtained in this research field. It is hoped that new research on this topic will not only generate new insights into the pathophysiology of miRNA in I/R injury but also can provide a basis for the clinical application of miRNA in I/R.

## 1. Introduction

Mature microRNA (miRNA) is a small but noncoding single-stranded RNA molecule with mature transcripts of 18-25 nucleotides. It acts as a negative regulator of gene expression by combining with the 3′-untranslated region (UTR) of complementary or partially complementary target messenger RNAs (mRNAs) to inhibit or degrade its expression. As a new gene expression regulation mechanism, its importance as a noncoding regulatory RNA is extensively studied [1]. miRNAs can participate in a variety of biological processes, such as gene transcription, posttranscriptional processing, cell differentiation, ontogeny, heredity, and epigenetics [2]. Notably, key roles have emerged in pathophysiological fields such as ischemia-reperfusion (I/R) injury [3].

## 2. miRNA Biosynthesis and Function

miRNAs are believed to be transcribed from DNA, and although it cannot be translated, it can regulate gene expression by changing the translation efficiency and/or stability of targeted mRNAs after transcription [4]. The initial transcript (primary miRNA; pri-miRNA) of miRNA is a long RNA transcript containing at least one hairpin-shaped miRNA precursor, which has a short existence time. It is cut by Ribonuclease III (RNase III) endonuclease and/or splicing elements similar to Drosha to form a 60-70 nt pre-miRNA with stem ring structure [5]. This structure is then transported from the nucleolus into the cytoplasm by Exportin-5, a guanosine triphosphate- (GTP-) bound form- (RanGTP-) dependent double-stranded RNA (dsRNA) binding protein that mediates the nuclear output of miRNAs [6]. In the cytoplasm, Dicer-like endonuclease cuts pre-miRNA into a double-stranded 18~25 nt mature miRNA and unwinds the chain into strand of miRNA and a miRNA passenger chain (miRNA∗). In one instance, the miRNA chains combine with the RNA-induced silencing complex (RISC), whereas the miRNA∗ is subsequently degraded. The miRNA/RISC complex combines with specific target sequence mRNA in a partially complementary manner, especially through base pairing in the 3′-UTR of mRNA, which leads to mRNA transcription inhibition or elimination, thus inhibiting protein expression [7].

**Figure 1:**
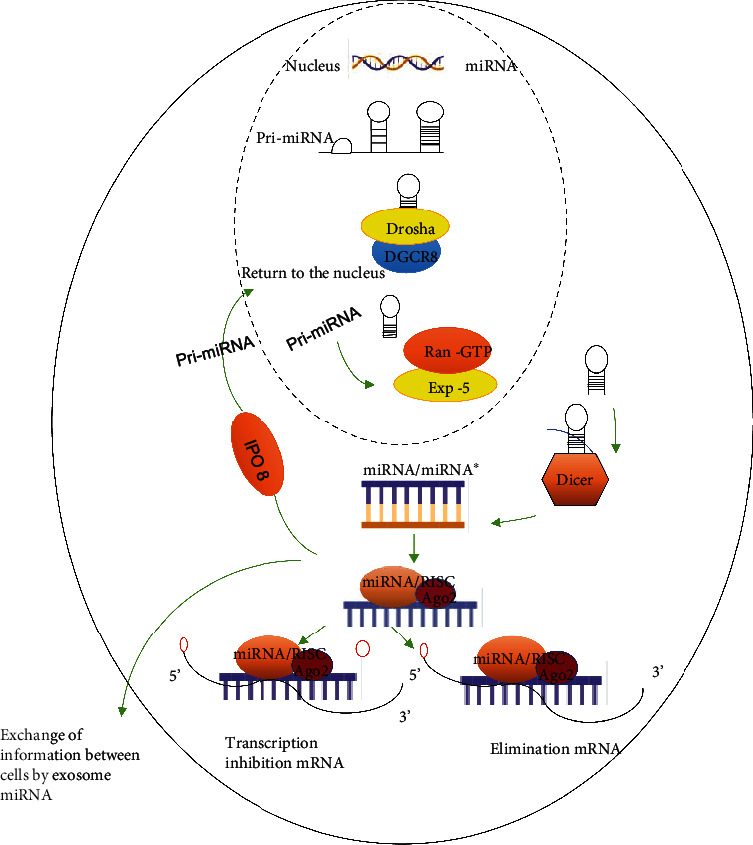
miRNA biosynthesis processing.

miRNAs can regulate gene expression, depending on their ability to complement one or more mRNA partial sequences, which are generally in the 3′-UTR region. This binding of miRNAs to target mRNAs can inhibit protein translation. In some cases, dsRNA is formed by binding to miRNAs, thus triggering the degradation of mRNAs [8]. However, in other cases, miRNA complexes only inhibit protein translation machines but can also inhibit protein translation by other methods that do not degrade RNA [9]. Most miRNAs can be found in the cytoplasm. However, increasing evidence shows that some mature miRNAs can reenter the nucleus and regulate the biological occurrence of other miRNAs or even their own expression [10]. The mediated nuclear transport of mature miRNAs is also dependent on the association of importin-8 (IPO8) with the argonaute-2 (Ago2) complex which determines the RISC-carrying miRNA chain transport to the nucleus [11]. In addition, the exogenous gene miRNAs regulate the expression of target genes, establish cell-to-cell communication, and participate in regulating different biological processes. Furthermore, exon-shuttle miRNAs and other RNAs are transported into cells without being degraded by extracellular RNAs. Montecalvo et al. [12] found that the transfer of nano-ovum (<100 nm) obtained from the intracellular pathway is a new communication mechanism between dendritic cells, while the transfer mechanism of exogenous shuttle miRNAs between dendritic cells serves a role in communication and posttranscriptional regulation (Figure 1).

Because the function of most miRNA inhibitory genes depends on partial complementation, a single miRNA can target multiple mRNA, while multiple miRNAs can also act on a single mRNA, thus cooperatively regulating the expression intensity of genes in different tissues and cells. Therefore, miRNAs may have extensive fine-tuning effects on protein-coding genes. Undoubtedly, the discovery of miRNAs has changed our understanding of gene regulation in the postgenome era.

## 3. miRNAs and I/R Injury

**Figure 2:**
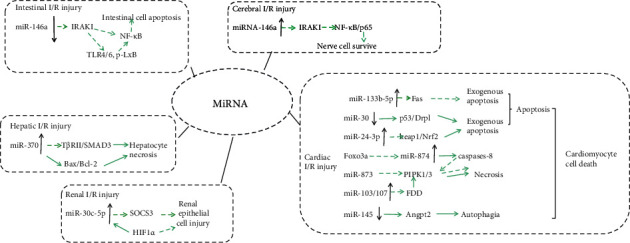
miRNAs in cerebral, intestinal, hepatic, cardiac, and renal I/R injury. Solid arrow indicates promote. Dotted arrow indicates inhibit.

I/R is a pathological state characterized by reduced tissue blood supply, accompanied by adverse reactions such as metabolite accumulation, vascular permeability [13], interstitial edema [14], and endothelial dysfunction [15] during ischemia, followed by the recovery of perfusion and accompanied reoxygenation, which increases the sensitivity of tissues in ischemia-induced hypoxia to oxygen after recovery of circulation, resulting in the activation of inflammatory factors and excessive autophagy. Furthermore, increases in reactive oxygen species (ROS), endothelial injury, and complement activation result in secondary injury to ischemic tissue and I/R injury [16]. I/R injury leads to some problems, including acute myocardial infarction (AMI) [17], ischemic stroke (IS) [18], acute kidney injury (AKI) [19], trauma [20], circulatory arrest [21], sickle cell disease, and sleep apnea [22]. In addition, I/R injury is a major problem in organ transplantation and general surgery [23]. Some evidences show that miRNAs are involved in the regulation of many pathophysiological processes of I/R injury with significant differential expression; these small RNAs are becoming promising therapeutic targets for ischemic diseases (Figure 2).

### 3.1. Myocardial Ischemia/Reperfusion Injury

Early reperfusion after AMI is an effective treatment method, but which may lead to myocardial cell dysfunction [24]. Therefore, it is prudent to reveal new molecular targets and networks involved in triggering and resisting myocardial I/R injury pathophysiology. Some studies have suggested that miRNAs are involved in this process. Dong et al. [25] used microarray analysis to investigate the expression characteristics of miRNAs in rat hearts after 6 h of I/R in early AMI. The results showed that among the 341 mature miRNA sequences, 38 miRNAs were differentially expressed in infarcted regions (21 up and 17 downregulated), and 33 miRNAs were abnormally expressed in marginal regions compared with noninfarcted regions. Specifically, miR-21, which is present in cardiac progenitor cell-derived exosomes, is significantly downregulated in infarcted areas and upregulated in border areas. Previously, we used denovirus-mediated miR-21 gene transfer technology, further confirmed the protective effect of miR-21 on myocardial cell injury caused by ischemia [26]. Overexpression of miR-21 significantly reduced the necrotic area of the heart after I/R and reduced the necrotic volume of the myocardium, thus leading to long-term remodeling of the heart and reduction of apoptosis. This was the first time that multiple miRNAs were confirmed to have abnormal expression in the early (6 h) border and infarcted area of AMI. However, it is still necessary to reveal the complex role of miRNAs in the pathophysiological process of AMI through computational analysis, prediction, or depletion experiments.

The fatty acid synthase (Fas) death pathway is considered an important medium for myocardial cell death and AMI during myocardial ischemia reperfusion injury (MI/RI) [27, 28]. The latest research by He et al. [29] used microarray analysis technology to show miRNAs differentially expressed after morphine preconditioning treatment. The results show that overexpression of miR-133b-5p increased cell activity, inhibited lactate dehydrogenase activity, and inhibited Fas gene and protein expression, thus reducing cell apoptosis and alleviating hypoxia/reoxygenation (H/R) intrusion. However, the *in vitro* data provided by miR-133b-5p mimetics and inhibitors may not fully replicate the *in vivo* environment and cannot fully prove that miR-133b-5p/Fas plays a key role in cardiac protection against I/R injury mediated by morphine pretreatment. Simultaneously, miR-133b-5 may also have a “one-to-man” target gene regulation mechanism, and further relevant pathways should therefore be explored.

### 3.2. Brain Ischemia/Reperfusion Injury

Rapid reconstruction of occluded blood vessels and early reperfusion to limit cerebral ischemia injury is an important stroke treatment strategy [18, 30]. However, this approach also leads to I/R injury. In recent years, the expression of miRNAs in the brain tissues has received considerable attention. The basic biological research process of miRNAs in IS has been rapidly carried out and is expected to be upgraded. Min et al. [1] found that miRNA expression profiles changed significantly, with 15 miRNAs upregulated and 44 miRNAs downregulated in brain I/R injury. The target genes of these differentially expressed miRNAs are mainly involved in metabolic and cellular processes and are recognized as pivotal nodes of miRNA-gene ontology (GO) networks. Another study also found that miR-21 and miR-29b both have upregulation effects in hypoxic and glucose-deficient neurons and astrocytes, and these neurons and astrocytes simulate I/R *in vitro* [31]. Both studies emphasized the role of miRNAs in the pathogenesis of brain I/R injury. However, the exact role and regulatory mechanism of miRNAs in brain I/R injury remain unclear.

A recent study reported that miR-146a is upregulated in the brain tissue of mice with experimental cerebral I/R injury. Inhibition of miR-146a results in upregulation of expression of interleukin receptor-related kinase-1 (IRAK1). IRAK1 aggravates neurobehavioral disorders caused by I/R, which expands the infarct scope, aggravates blood-brain barrier leakage, and intensifies brain I/R-induced neuronal cell death by activating the nuclear factor kappa B (NF-*κ*B) signaling pathway and nuclear accumulation of p65. Therefore, we hypothesize that miR-146a may be a potential target for the brain I/R injury treatment [32].

### 3.3. Hepatic Ischemia/Reperfusion Injury

Hepatic I/R injury is a common complication of liver transplantation and general anesthesia [33]. miR-370 is a marker with important functions in several human tumors, including gastric cancer [34], liver cancer [35], prostate cancer [36], and laryngeal squamous cell carcinoma [37]. In recent years, researchers have found that miR-370 is also a promising noninvasive biomarker for patients with liver I/R injury. Li et al. [38] found that miR-370 levels increased after liver I/R. Inhibition of miR-370 can reduce the expression of serum transaminase and proinflammatory cytokines and improve histological damage in the liver. They also confirmed that this mechanism may be linked to miR-370 targeting of the 3′-UTR of transforming growth factor-*β* receptor II (TGF*β*RII) with a potential role in hepatic I/R injury. Meanwhile, Zare et al. [39] found that bone marrow-derived mesenchymal stem cell (BM-MSCs) had a stronger effect on liver I/R mice than ischemic preconditioning. In addition, in BM-MSCs, downregulating the inhibitory effect of miR-370 on liver injury may play a role by decreasing the expression of Bax and upregulating Bcl2. However, the exact regulatory mechanism of miR-370 in hepatic I/R injury is still not fully understood, and further research is needed to clarify the mechanism. Interestingly, the expression level of miR-370 also has a protective effect against MI/RI [40]. Therefore, it can be concluded that the effect of miR-370 is stimulus- and cell/tissue-type dependent.

### 3.4. Renal Ischemia/Reperfusion Injury

Renal I/R injury may be related to surgical treatment and is a common phenomenon in kidney transplantation [41, 42]. On one hand, renal injury caused by I/R, especially acute renal failure, is closely related to hypoxia and inflammatory reaction [43] which promotes endothelial cell activation, injury, and leukocyte adhesion, triggers leukocyte clamping, and damages microvascular blood flow. On the other hand, the initial nonimmune hypoxia injury and subsequent reperfusion will also activate the natural immune response, resulting in different degrees of tissue damage. The prognosis of AKI is poor because there is no effective treatment or prevention of I/R-induced AKI. Therefore, there is an urgent need to develop an effective method to treat AKI caused by I/R. Study has shown that preventing the execution of mitochondrial fission and maintaining mitochondrial homeostasis is a potential treatment [44]. miRNAs are important regulators of gene expression. They can silence genetic information after transcription by targeting the 3′-UTR of mRNAs and eventually inhibit protein synthesis or increase mRNA degradation.

Wang et al. [45] detected miRNAs in the kidney 12 h after renal I/R injury with an miRNA chip and determined that 36 miRNAs were abnormally expressed in the kidneys of rats with renal I/R injury, of which 15 miRNAs showed significant change. Quantitative real-time PCR results were also consistent with the chip results. Notably, miR-10a, miR-192, and miR-194 in the plasma of rats with renal I/R injury were significantly increased, of which miR-10a was increased within 1 h of reperfusion, and the peak time of miR-192 and miR-194 is similar to that of serum creatinine 6 h after reperfusion. Therefore, miR-10a in plasma may be the best biomarker for renal I/R injury in rats.

Zou et al. [46] quantitative determination of miR-30c-5p, miR-378a-3p, and miR-192-5p levels in the urine of rats and AKI patients confirmed miR-30c-5p and miR-192-5p as possible diagnostic markers of I/R-induced AKI. Previous studies have shown that hypoxia inducible factor-1*α* (HIF1*α*) is a key transcription factor that protects cells from hypoxic injury [47, 48]. miR-30c-5p is upregulated in an H/R model and can protect epithelial cells from anoxic damage, whereas HIF1*α* obviously promotes this protective effect. In addition, miR-30c-5p stabilizes the expression level of HIF1*α* by targeting suppressor of cytokine signaling-3 (SOCS3) [46]. Some studies have confirmed that renal I/R injury can activate critical inflammatory cascades, including activation of NF-*κ*B [49, 50]. In the process of renal I/R injury, oxidative stress and inflammatory reactions are manifested by activation of the HIF1*α* hypoxia and NF-*κ*B inflammation signaling pathways, whereas transcription of most inflammatory factors and adhesion factors are regulated by both of these. A study showed that HIF1*α* and NF-*κ*B have synergistic effects in the treatment of AKI [51]. Since the NF-*κ*B signaling pathway is an important target for miRNAs to respond to renal I/R, elucidating whether NF-*κ*B activity is regulated by miR-30c-5p could be of interest.

### 3.5. Intestine Ischemia/Reperfusion Injury

Intestinal I/R injury is an important problem in abdominal aortic aneurysm surgery, small bowel transplantation, extracorporeal circulation, strangulated hernia, and neonatal necrotizing enterocolitis [52]. Among the pattern recognition receptors, toll-like receptor (TLR) is the main sensor that produces an inflammatory network and is also a key regulator of the natural immune system [53]. Uncontrolled activation of the natural immune system by TLR plays a key role in I/R-mediated tissue injury. Tumor necrosis factor-*α* (TNF*α*) and interleukin-1*β* (IL-1*β*) are the two most effective cytokines secreted during TLR ligand stimulation of the natural immune activation process [54]. IL-1*β* signaling induces the NF-*κ*B signaling pathway through activation of myeloid differentiation primary response-88 (MyD88), IL-1R-related kinases (IRAK1, IRAK2, IRAK4), TNF-related associated factor-6 (TRAF6), and TGF*β*-activated kinases, thus immediately and temporarily activating normal physiological responses to pathogens.

miR-146a is considered to have a negative effect on the innate immune response [55, 56]. Chassin et al. [57] used mouse intestinal I/R injury models and human intestinal mucosa biopsy to observe the enhanced expression of the TLR signaling molecule IRAK1 protein in ischemic epithelial cells and found that its reactivity to natural immune stimulation was significantly enhanced. When miR-146a is induced by 3,3′-diinodolylmethane (DIM), it controls the level of epithelial IRAK1 protein in mouse and human mucosal tissues through translation inhibition and protection of I/R injury. This indicates the application or pharmacological induction of miR-146a as a new strategy to control natural immune hyperreactivity and reduce transient hypoxia or tissue injury after I/R.

miR-146a improves the survival of intestinal epithelial cells under ischemia and I/R injury by inhibiting TLR4, TRAF6, and phosphorylation of NF-*κ* light polypeptide gene enhancer in B-cells inhibitor-*α* (p-I*κ*B*α*), thus leading to a decrease in nuclear translocation of NF-*κ*B p65, an increase in cell survival rate, and a decrease in cell apoptosis [58].

## 4. Diagnostic Biomarkers of I/R

In the past, endothelial cell injury and troponin have been used as markers of I/R injury [59, 60], but the progression and prognosis of the disease cannot be accurately determined by the degree of endothelial cell injury, while the delayed release time of troponin often leads to low sensitivity. Recently, differential changes in miRNAs in I/R injury have been confirmed. Given the specificity, timing, and relative conservativeness of miRNA expression, miRNAs have great potential as biomarkers. miRNAs showed high conservation in sequences of different species, which promotes the translation from animal models to humans. miRNAs have high tissue specificity, which is an obvious imbalance in many human diseases [61]. In addition, they are stable in tissues and body fluids (such as plasma, serum, and exosomes), which make miRNAs suitable as repositories for biomarker discovery.

Rensen et al. [62] performed miRNA sequence analysis using microarray analysis and quantitative polymerase chain reaction (qPCR) techniques in groups of patients with either acute ischemic stroke (AIS) (10 cases) or neurological diseases (10 cases). A total of 183 different miRNAs were detected in the cerebrospinal fluid, of which two miRNAs (let-7c and miR-221-3p) were both associated with stroke. A total of 287 different miRNAs were detected in the blood, of which two miRNAs (miR-151 a-3p and miR-140-5p) were upregulated and one miRNA (miR-18b-5p) was downregulated in the stroke group.

Xue et al. [63] proved for the first time that miR-26a-1, miR-146, and miR-199a-1 can be used as candidate biomarkers for AMI. The expression levels of miR-26a-1, miR-146, and miR-199a-1 in AMI patients were significantly higher than those in normal individuals. Meanwhile, the expression levels of three kinds of miRNAs in AMI patients before and after percutaneous coronary intervention (PCI) were significantly higher than those in the control group. The combined detection of three kinds of miRNAs had higher AUC values before and after PCI, suggesting that the combined application of miRNAs has higher accuracy in the diagnosis of AMI.

No miR-519e-5p was detected in the plasma of healthy volunteers and non-AMI patients (IS patients and pulmonary embolism (PE) patients), but the levels of three circulating miRNAs in IS and PE were all increased, while the plasma miR-519e-5p level was only detected in AMI [64]. Circulating miR-21-5p and miR-361-5p (especially miR-361-5p) showed an upward trend similar to that of plasma cardiac troponin I (cTnI) in early AMI (0-4 h). Circulating miR-519e-5p levels are relatively low in AMI patients, which is contrary to the expression in other ischemic diseases. These results indicate circulating miR-519e-5p as an effective biomarker for distinguishing acute AMI from PE and IS. Recently, a study showed that miR-423-5p can be used as a useful biomarker for clinical ischemic diseases. Rizzacasa et al. [65] reported that miR-423-5p may be related to the risk and severity of either stable or unstable coronary heart disease (CHD) 6 months after AMI. miR-423-5p provides a new genetic marker for the early prevention and intervention of CHD.

Schulte et al. [66] proved that miR-126, miR-197, and miR-223 derived from serum participate in intravascular inflammation and platelet activation and may be considered as biomarkers of cardiovascular death in patients with CHD. Jickling et al. [67] compared the levels of miRNAs between the peripheral blood of 24 patients with IS and 24 patients with risk factors. Microarray analysis showed that the expression levels of six miRNAs were downregulated (miR-122, miR-148A, let-7i, miR-19a, miR-320d, miR-4429), and two were upregulated (miR-363 and miR-487b).

Zhou et al. [68] reported that high levels of miR-134 can be detected in serum samples of patients with AIS within 24 h, which may be suitable as a potential genetic marker of the disease. Zhang et al. [69] found that the plasma miR-192 levels in patients with AKI were significantly higher than those in patients without AKI 2 h after admission. It is suggested that the dynamic change of miR-192 in plasma levels in patients with AKI may be a predictor of early ischemic AKI.

In summary, miRNAs associated with I/R injury are promising biomarkers for identifying ischemic diseases and assessing their prognosis (Table 1). With the development of qPCR, miRNAs have been widely used in the translation field [70]. The I/R-related diseases discussed above were not diagnosed at the onset of a large number of studies; the diagnostic value of these miRNAs remains to be determined in future. Therefore, to apply these miRNAs in clinical practice, clinical trials with large sample sizes and long-term follow-up are needed.

## 5. Targeted miRNAs Treating I/R Injury

Studies have found that correcting the expression of one or several key genes in I/R injury can restore the physiological expression levels of damaged genes, which provides an opportunity for the molecular-targeted therapy of ischemic diseases. As a key regulator of gene expression, miRNAs not only open up a new research field for biomarker research of ischemic diseases but also for the treatment of ischemic diseases [71]. To date, several methods have been developed to down- or upregulate miRNAs, among which miRNA mimetics and inhibitors have shown promise as new therapeutic drugs in clinical application [72].

### 5.1. Anti-miRNA Oligodeoxynucleotide Technology

The most effective inhibitor of miRNA is anti-miRNA oligonucleotides (AMO), which can combine with miRNA, inhibit the combination of endogenous miRNA and mRNA, and relieve the inhibitory effect of miRNA on mRNA. AMO is unstable and easily degraded *in vivo*; therefore, various chemical modifications are used to improve the stability of AMO *in vivo*. These include locked nucleic acid (LNA), 2′-fluoro-RNA (2′-F-RNA), 2′-O-methoxyethyl (MOE), peptide nucleic acid (PNA), and phosphorodiamidate morpholino oligonucleotides (PMOs). Properly designing a specific AMO can improve the performance and efficiency of targeting miRNAs by increasing nuclease resistance and target affinity [73].

AMO can be a potential therapeutic method by inhibiting disease-related miRNAs. In the treatment of I/R-related diseases, ischemic postconditioning (IPost) can downregulate relevant miRNAs to protect the heart from infarction after reperfusion. Tu et al. [74] introduced 2-O methyl AMO complementary to miR-21 sequence into mouse myocardium and found that using Antagomir-21 instead of an interfering agent can reduce the protective effect of post on mouse MI/RI. In addition, after reperfusion for 3h, Antagomir-21 drug-treated hearts showed a significant decrease in cardiac function recovery, showing a decrease in left ventricular ejection fraction (EF) and fractional shortening (FS) and an increase in left ventricular end diastolic diameter (LVEDD) and left ventricular end-systolic diameter (LVESD).

Shen et al. [75] found that the infusion of LNA-miR-30 family inhibitor can correspondingly increase the levels of cystathionine-gamma-lyase (CSE) and hydrogen sulfide (H_2_S) after AMI, reduce the infarct area, reduce the number of apoptotic cells in the infarct area, and improve cardiac function after AMI. There are also reports of *in vivo* studies, using intravenous injection or tissue local injection, that evaluated the feasibility and effectiveness of miRNA application as a treatment.

### 5.2. siRNA Gene Knockout Technology

Small interfering RNA (siRNA) is a chemically synthesized dsRNA with a length of 19-23 nt. To function, an siRNA must be absorbed by specific cells and enter the cytoplasm. siRNAs are contained in RISC and transferred to the Argonaute protein of the RISC catalytic core. Protein-RNA complexes separate dsRNA and discard the passenger strands [76, 77]. Then, RISC, guided by the antisense strand, downregulates the expression of precise genes by either degrading specific mRNA or physically blocking mRNA translation [78, 79].

siRNA-mediated silencing has been proven to be effective in targeting miRNAs of interest in mice. For example, siRNA knockout of lethal-7 (let-7) can effectively inhibit the expression of let-7 in nerve cells, thereby reducing apoptosis and neurological deficits after cerebral I/R injury [80]. A recent study showed that mice treated with necrosis-related factor (Nrf) siRNA showed a decrease in the AMI area and an improvement in cardiac function during MI/RI [81]. These studies show that downregulation of miRNAs through siRNAs may be a promising tool for the treatment of ischemic diseases.

### 5.3. miRNA Sponge Technology

miRNA sponges are RNA molecules with a plurality of miRNAs in series, which can separate the required miRNAs from the target miRNAs [82]. Endogenous miRNA sponges, also known as competing endogenous RNAs (ceRNAs), act to buffer the activity of miRNAs on physiologically related targets. Its principle is dominant negative regulation, and it can efficiently inhibit miRNA action for a prolonged time [83]. The application of miRNA sponges in the treatment of ischemic diseases has only just begun. Li et al. [84] infected miRNA sponges with miR-497 binding site and adenovirus with recombinant plasmid into neonatal rat cardiomyocytes (NRCS) under aortic regurgitation (AR) and left ventricular myocardium of mice under I/R. Studies have found that miRNA sponges inhibiting miR-497 can upregulate the expression of the antiapoptosis gene *Bcl2* and autophagy gene *LC3B*, significantly reduce cell apoptosis, and enhance autophagic flux. In addition, the effectiveness of miRNA sponges can be evaluated by detecting the expression of the target genes with a fluorescence reporting system. Generally, if there is a target miRNA, the fluorescence reporting system should be significantly inhibited by miRNA sponges.

At present, miRNA sponges are mainly used in the experimental field of miRNA function research, while their clinical application still needs further evaluation. miRNA sponge technology has many advantages, including equivalent efficacy to AMO, and is also simpler than gene knockout with application in various cell lines and models. Furthermore, miRNA sponges only affect mature mRNAs and can inhibit the action of a specific miRNA without affecting other miRNAs. However, in the field of ischemic disease treatment, antisense technology may be more promising pending the continuous development and improvement of antisense technology and transfer of oligonucleotides into cells.

### 5.4. Peptide Nucleic Acid Technology

PNA is a synthetic analog of DNA, which was introduced by Nielsen et al. [85] in 1991. Anti-miRNA PNA can bind miRNAs through steric hindrance and inhibit their interaction with RISC, thereby reducing the expression of miRNAs [86]. PNA has a number of advantages over other anti-miRNA substances, such as not having to target full-length miRNAs. Thus, PNA is a good candidate for biomedical applications [87]. Studies have shown that PNAs have the ability to efficiently inhibit miR-155, which is expressed in the hematopoietic system in primary B cells as well as in a mouse model of lymphoma [88, 89]. A study has shown that intraocular injection of antisense vascular endothelial growth factor (*Vegf*) PNA or antisense Ephrin type-A receptor 2 (*EphA2)* PNA can significantly reduce their respective target mRNAs in ischemic retinas and inhibit retinal neovascularization [90]. Downregulation of miRNA by PNA is expected to be an effective intervention for ischemic diseases. However, more data are needed on its potential side effects or toxicity.

### 5.5. miRNA Overexpression Vectors

In recent years, viral transfection (including lentivirus and adenovirus) has continued to occupy a central position in gene therapy as transgenic vectors, with high transfection efficiency. Using different viral vector tools can realize efficient and stable transduction of cells and animals and gradually become a substitute for miRNA overexpression plasmids. For example, Qu et al. [91] found that the increased overexpression of miRNA-126 promoted cerebrovascular generation and neurogenesis, which further improved neurobehavioral results in ischemic mice. Wang et al. [92] administered lentivirus carrying miR-214 to reduce the area of AMI after I/R injury by targeting the expression of Bri1-Ems-Suppressor1- (BES1-) interacting Myc-like protein-1 (Bim1) and preventing its transfer from cytoplasm to mitochondria *in vivo* and *vitro*. This significantly reduced the apoptosis of myocardial cells induced by I/R and further protected the heart from I/R injury without toxicity.

Overexpression of traditional viral vectors is usually systemic rather than specific to target cells or tissues. However, various adenoviral vector systems have shown improve targeting for gene delivery, which are expected to be used in gene therapy and treatment of diseases related to I/R. These vectors are easy to handle, have a wide range of tropism, and have a high titer capability. Furthermore, they have been optimized to avoid unnecessary side effects and target specific tissues or cell types [93].

### 5.6. miRNA Mimetics

miRNA mimetics are small, chemically modified dsRNAs that mimic mature miRNAs *in vivo*, which have been widely used in functional experiments. Selvamani and Sohrabji [94] successfully inhibited the expression of caspase-3 in neuronal cells of female stroke patients through synthetic miR-363 mimetics targeting overexpressed caspase-3 protein. Although miR-363 therapy has a strong neuroprotective effect on middle-aged women, it does not reduce the infarct volume of men or improve sensorimotor function. The mi363-3p mimetic is located in neurons and may reduce the infarct volumen by influencing on the apoptosis process. miR-144 has strong acute myocardial protection in I/R injury models. Zhou et al. [95] proved that intravenous injection of miR-144 mimetics has a strong effect on remodeling after AMI, which indicate that miR-144 has a potential therapeutic effect after AMI.

Many miRNA mimetics, such as pre-miRNA precursor (Ambion) and miRIDIANTM microRNA mimetic (Thermo Scientific Dharmacon), have been commercialized. Because miRNA mimetics have no carrier toxicity, if the drug delivery system was verified to have no side effects after long-term use, it will be a promising therapy for anti-ischemic diseases.

## 6. Discussion

As a relatively new research field in ischemic diseases, miRNAs have attracted attention with broad application prospects. The therapies based on miRNA have achieved remarkable results in various I/R injury in vivo and in vitro. The role of miRNAs in diagnosis, changes, and prognosis of different types of ischemic diseases is exciting. It is noteworthy that the expression level of miRNAs in plasma, serum, and blood cells in patients with ischemic diseases has changed greatly, which emphasizes the potential role of miRNAs in the diagnosis and treatment of ischemic diseases. With further in-depth study about the mechanism of miRNAs in the occurrence and development of diseases, some treatment methods based on miRNA will change human disease treatment dramatically. Once the problems and challenges of miRNAs in the ischemic disease treatments are solved, miRNAs will become one of prominent candidates competing with protein inhibitors. However, some technical and practical difficulties still need to be solved before they can be converted into clinical practice.

Increasing evidence shows that miRNAs are extensively involved in I/R injury. For instance, miRNAs play a crucial role in I/R injury and regulation of the inflammatory NF-*κ*B signaling pathway. The lack of conservativeness of miRNA sequences between different species in basic research studies is still an obstacle to its clinical application. These miRNA-related therapies are effective in humans, and its potential adverse reactions remain to be answered. In addition, the regulation of miRNAs on target genes affects their transcription or degradation, as well as the regulation of other expressed miRNAs after returning to the nucleus, and the functions of exogenous miRNAs in cell-to-cell communication may be different between animals and humans. Therefore, the in-depth preclinical research should be performed on targeted diseases to determine their functional characteristics before patients receive treatment.

In summary, this study highlighted the broad applications of miRNAs I/R injury and elaborated on targeted therapies using gene expression regulation methods. These included antisense oligonucleotides and siRNA gene knockout techniques, miRNA sponges, PNA techniques, miRNA overexpression vectors, and miRNA mimics which interfere with miRNA expression and their application to treat I/R injury. Currently, many of these technologies have shortcomings with stability and delivery as the most common problems, which are potential reasons why miRNAs have not yet reached clinical translation. Only when miRNAs are reasonably designed and combined with existing clinical treatment methods can more effective disease treatment schemes be formed. This review provided new research results obtained in this research field. It is hoped that new research on this topic will not only generate new insights into the pathophysiology of miRNA in I/R injury but also can provide a basis for the clinical application of miRNA in I/R. There is no doubt that pursuing this course of treatment will be long, making it challenging. Therefore, it is prudent to continue to explore miRNAs as potential therapies for I/R injury.

## Figures and Tables

**Table 1:** miRNAs associated with I/R injury as a clinical biomarker of ischemic diseases.

miRNA	Disease	Cases	Controls	Source	Reference
let-7c, miR-221-3p, miR-151a-3p, miR-140-5p, miR-18b-5p	AIS	20	10	Cerebrospinal fluid blood	[62]
miR-26a-1, miR-146a, miR-199a-1	AMI	58	27	Plasma	[63]
miR-519e-5p	AMI	45	28	Plasma	[64]
miR-423-5p	CHD, AMI	99	38	Plasma, PBMCs	[65]
miR-126, miR-197, miR-223	CHD	873	340/533	Serum	[66]
miR-134	AIS	100	50	Serum	[68]

## Abbreviations

2′-F-RNA: 2′-Fluoro-RNA
Ago2: Argonaute-2
AIS: Acute ischemic stroke
AKI: Acute kidney injury
AMI: Acute myocardial infarction
AMO: Anti-miRNA oligonucleotides
AR: Aortic regurgitation
Bax: Bcl2-associated X protein
Bcl2: B-cell lymphoma-2
BES1: Bri1-Ems-Suppressor1
Bim1: BES1-interacting Myc-like protein-1
BM-MSC: Bone marrow-derived mesenchymal stem cell
CHD: Coronary heart disease
CBA: Chicken *β* -actin
ceRNA: Competitive endogenous RNA
CSE: Cystathionine-gamma-lyase
cTnI: Cardiac troponin I
DIM: 3,3′-Diinodolylmethane
dsRNA: Double-stranded RNA
EphA2: Ephrin type-A receptor 2
EF: Ejection fraction
Fas: Fatty acid synthase
FS: Fractional shortening
H/R: Hypoxia/reoxygenation
H2S: Hydrogen sulfide
HIF1*α*: Hypoxia inducible factor-1*α*
IL-1*β*: Interleukin-1*β*
IPO8: Importin-8
IPost: Ischemic postconditioning
IRAK: Interleukin-1 receptor-associated kinase
IS: Ischemic stroke
LC3-II: Light chain 3-II
let-7: Lethal-7
LNA: Locked nucleic acid
LVEDD: Left ventricular end diastolic diameter
LVESD: Left ventricular end-systolic diameter
MI/RI: Myocardial ischemia reperfusion injury
miRNA: MicroRNA
miRNA∗: MicroRNA passenger chain
MOE: 2′-O-methoxyethyl
MyD88: Myeloid differentiation primary response-88
NF-*κ*B: Nuclear factor kappa B
NRCS: Neonatal rat cardiomyocytes
Nrf: Necrosis-related factor
PE: Pulmonary embolism
p-I*κ*B*α*: Phosphorylation of NF-*κ* light polypeptide gene enhancer in B-cells inhibitor-*α*
PCI: Percutaneous coronary intervention
PMO: Phosphorodiamidate morpholino oligonucleotide
PNA: Peptide nucleic acid
pre-miRNA: miRNA precursor
pri-miRNA: Primary miRNA
qPCR: Quantitative polymerase chain reaction
RISC: RNA-induced silencing complex
ROS: Reactive oxygen species
siRNA: Small interfering RNA
SOCS3: Suppressor of cytokine signaling-3
TGF*β*RII: Transforming growth factor-*β* receptor II
TLR: Toll-like receptor
TNF*α*: Tumor necrosis factor-*α*
TRAF6: TNF receptor associated factor-6
UTR: Untranslated region
Vegf: Vascular endothelial growth factor.
